# Mangiferin mitigates neurological deficits and ferroptosis via NRF2/ARE pathway activation in cerebral ischemia-reperfusion rats

**DOI:** 10.3389/fphar.2025.1577954

**Published:** 2025-05-22

**Authors:** Chenjia Peng, Yang Zhang, Jiaqing Chai, Hengbo Zhang

**Affiliations:** ^1^ School of Mathematics and Statistics, Hunan First Normal University, Changsha, China; ^2^ School of Physical Education, First Hunan Normal University, Changsha, China

**Keywords:** MGF, ischemic stroke, ferroptosis, Nrf2/ARE pathway, neuroprotection

## Abstract

**Introduction:**

Ferroptosis is a newly described form of nonapoptotic, iron-dependent cell death that plays an essential role in the pathogenesis of ischemic stroke. Targeting ferroptosis may be an effective way to treat ischemic stroke. Mangiferin (MGF) is a natural polyphenol that has been shown to protect neurological function via multiple mechanisms. However, the mechanism by which MGF inhibits ferroptosis in stroke remains unclear.

**Methods:**

An ischemic stroke rat model was established by middle cerebral artery occlusion. Neurological scoring, TTC staining, behavioral tests, Nissl staining, HE staining and immunochemistry were used to analyze the influences of MGF on neurological deficits, the infarct area, cognitive function, neuronal morphology, histopathological injury, and the morphology of microglia and astrocytes. Transmission electron microscopy and Perls’ stain were used to evaluate the characteristics of ferroptosis. Western blotting was used to analyze the expression of NRF2, FTL, SLC7A11 and GPX4. ELISA was used to analyze the levels of cytokines, including IL-6, IL-1β and TNF-α, to evaluate neuroinflammation. Oxidative stress was evaluated by analyzing the levels of ROS, MDA, GSH, and SOD.

**Results:**

MGF clearly improved the neurological function and learning and memory ability of stroke rats. MGF significantly decreased ROS and MDA and increased GSH, SOD. MGF significantly suppressed neuroinflammation by downregulating IL-6, IL-1β and TNF-α. Ferroptosis in stroke rats was significantly inhibited by MGF. MGF significantly increased the expression of NRF2, FTL, SLC7A11 and GPX4. The NRF2 inhibitor ML385 significantly reversed the effects of MGF on stroke rats.

**Conclusion:**

MGF protects neurological function and suppresses ferroptosis via activating NRF2/ARE pathway in ischemic stroke rats.

## 1 Introduction

According to the most recent Global Burden of Disease 2021 stroke burden estimates, stoke is still the third leading cause of death and disability (over 160 million) (GBD, 2021 Stroke Risk Factor Collaborators, 2024). Currently, reperfusion by thrombolysis is the mainstream treatment for ischemic stroke. Recombinant tissue plasminogen activator (rt-PA) is the only clinically approved thrombolytic drug. However, the use of rt-PA is limited by a short treatment window, increased risk of bleeding and aggravated reperfusion injury (Koscumb et al., 2024). Neuronal injury after ischemic stroke is a complex process involving various mechanisms, including excitatory amino acid release, Ca^2+^ overload, excess free radical production, inflammation, and programmed cell death (Zhou et al., 2018). Neuroprotective therapies that interrupt key ischemic cascades have shown promise but require further validation (Buchan and Pelz, 2022). It has been demonstrated that some traditional Chinese medicines have a protective effect on ischemic stroke with fewer side effects and act through multiple mechanisms, serving as an important resource for the development of potential neuroprotectants (Liu A. et al., 2023). Unfortunately, most neuroprotectants that have been effective in animal models have failed in clinical trials thus far.

Mangiferin (MGF) is a natural polyphenol widely distributed in many herbs and mango leaves. Previous studies have shown that MGF has a protective effect on neurological diseases, such as Parkinson’s disease, Alzheimer’s disease, depression, anxiety, and stroke (Hui et al., 2024). In ischemic stroke models, it has been demonstrated that MGF can improve neurological function by suppressing oxidative stress through the targeting of the NRF2/HO-1 (Li et al., 2019) and SIRT1/PGC-1α signaling pathways (Chen et al., 2021). Multiple mechanisms have been reported to be involved in the action of MGF, including antioxidative stress, anti-inflammation, immune regulation and programmed cell death modulation, such as ferroptosis (Feng et al., 2014). Ferroptosis is an iron dependent, nonapoptotic form of cell death characterized by intracellular lipid ROS accumulation and changes in mitochondrial morphology, including decreases in volume, increases in membrane density, and reductions in the number of mitochondrial cristae (Dixon and Olzmann, 2024). Ferroptosis is present along with apoptosis and necrosis in brain tissue samples from stroke patients (Li et al., 2018), indicating that ferroptosis may play an essential role in the pathogenesis of stroke and providing a potential target for stroke treatment. Recent data have shown that MGF is a potential ferroptosis inhibitor that can be used as a potential agent for the treatment of ferroptosis-associated diseases (Deng et al., 2024). However, whether MGF can inhibit ferroptosis in stroke to improve neurological function and the related mechanism of action is still unclear.

NRF2 is a redox-sensitive factor that activates downstream genes through binding to antioxidant response elements, and plays various roles, such as antioxidation, anti-inflammation, antiapoptotic, antiautophagic effects and protection of mitochondria (Zhang et al., 2019). Notably, the expression level of NRF2 is closely correlated with ferroptosis sensitivity. Genes related to ferroptosis, such as FTL, SLC7A11, and GPX4, are transcriptional targets of NRF2 (Zeng et al., 2023; Liu et al., 2020). MGF has been shown to play a therapeutic role in a variety of diseases, including kidney injury, tumors, heart ischemia-reperfusion injury and stroke, by activating NRF2 (Yang et al., 2016). Therefore, we hypothesized that MGF alleviates neurological disfunction and ferroptosis via activating NRF2/ARE pathway.

In this study, we investigated the protective effect of MGF on neurological function and cognition in stroke rats, and further explored the mechanism by which MGF in inhibits ferroptosis by activating the NRF2/ARE pathway.

## 2 Materials and methods

### 2.1 Animals

SD rats (male, 260–280 g) were obtained from Changsha Slac Laboratory Animal Co. Ltd. and kept in an environment with consistent diurnal rhythm changes (12/12 h light/dark alternation), humidity (50% ± 5%), and temperature (23°C ± 1°C) with free access to food and water. The animal protocol was approved by the ethics committee of First Hunan Normal University. Efforts were made to minimize the suffering of the animals.

### 2.2 Ischemic stroke model preparation, rat grouping and treatment

An ischemic stroke model was prepared by middle cerebral artery occlusion (MCAO). Briefly, the rats were anesthetized via an intraperitoneal injection of 30 mg/kg pentobarbital sodium. The common carotid artery, external carotid artery and internal carotid artery were isolated and exposed, and the common carotid artery was lapped. A filament (Sunbio Biotech, Beijing, China) was inserted into the middle cerebral artery. The animals were randomly divided into 4 groups: the sham group, the veh group, the MGF group, and the ML385+MGF group. The surgical procedure for the sham group is the same as those used for MCAO with the exception of inserting the filament into the middle cerebral artery. The rats in the veh group, MGF group and ML385+MGF group were respectively pre-treated with solvent, 20 mg/kg MGF and 20 mg/kg MGF combined with 15 μg/kg ML385 by intraperitoneal injection for 7 consecutive days before MCAO. ML385 was dissolved in 4% DMSO. The dosages of MGF and ML385 were selected according to previous studies (Hao et al., 2023; Hu et al., 2022). The subsequent animal experiments were performed by researchers who were blinded to the animal grouping. The sample size for every experiment was determined according to previous studies (Chen et al., 2022; Yan et al., 2021).

### 2.3 Neurological scoring

Neurological function was evaluated by an experimenter who was blind to the animal grouping according to the 5-point standard of Zea-Longa (Longa et al., 1989) 24 h after MCAO: normal, 0 points; the contralateral forepaw was not fully extended, 1 point; turning around to the left side, 2 points; falling and unable to stand, 3 points; inability to walk and loss of consciousness, 4 points; and death, 5 points.

### 2.4 TTC staining

TTC staining was performed by an experimenter who was blind to the animal grouping. The infarct area was evaluated by TTC staining 24 h after MCAO. The brain was quickly dissected and frozen for 30 min, after which 2 mm continuous coronal sections were prepared. The sections were immediately placed in a 2% TTC solution and incubated at 37°C for 30 min.

### 2.5 Nissl staining

The sections were immersed in Nissl staining solution, incubated at 56°C for 1 h and then washed with deionized water. The sections were differentiated in differentiation solution.

### 2.6 Behavioral tests

#### 2.6.1 Open field test

The open field test was performed by an experimenter who is blinded to the animal grouping. After 2 h of adaptation to the environment of the behavioral room for 2 h, the rat was placed in the central area of the open field and allowed to explore freely for 5 min. The total distance traveled by the rats was recorded. After each test, the rat was put back into its cage. The open field equipment was cleaned and disinfected.

#### 2.6.2 Novel object recognition test

The novel object recognition test was performed by an experimenter who is blinded to the animal grouping. The rat was placed in the box with two identical objects and allowed to explore freely for 10 min. After 2 h, one of the objects was replaced with a novel object with a different shape and color. The rat was allowed to move freely for another 10 min. The time spent by the rat exploring or sniffing the two objects was recorded. After each test, the rat was put back into its cage. The novel object recognition test equipment was cleaned and disinfected. The discrimination ratio was calculated according to the following formula: (Time _Novel_-Time _Familiar_)/(Time _Novel_ + Time _Familiar_).

### 2.7 Oxidative stress analysis

A total of 50 mg of cerebral cortex from the right hemisphere was homogenized and centrifuged and the supernatant was collected.

#### 2.7.1 ROS analysis

ROS analysis was performed by a ROS Assay Kit (O13 Red Probe) (catalogue no. HR9097, Baiaolaibo Technology, Beijing, China). A total of 190 μL of sample and 10 μL of O13 probe were mixed and added to a 96-well plate. The plate was incubated at 37°C for 30 min in the dark. The fluorescence intensity was measured at an excitation wavelength of 488 nm and an emission wavelength of 610 nm. The ROS level was calculated as fluorescence value per mg of protein and presented as the fold change compared with those in the sham group.

#### 2.7.2 MDA analysis

MDA analysis was performed according to the manufacturer’s instructions (catalogue no. A003-1-2, Jiancheng Bioengineering Institute, Nanjing, China). TBA solution (0.6%) was added to the homogenate supernatant, and the mixture was incubated in boiling water for 15 min. After rapid cooling, the mixture was centrifuged, and the absorbance value of the supernatant at 532 nm was determined. The MDA concentration was calculated according to the standard sample and expressed as nanomoles MDA per mg of protein (nmol/mgprot).

#### 2.7.3 GSH analysis

The GSH assay was performed according to the manufacturer’s instructions (catalogue no. D799614, Sangon Biotech, Shanghai, China). A total of 20 μL of sample was mixed with 140 μL of solution 2 and 40 μL of solution 3 and incubated for 2 min at room temperature. The absorbance at 412 nm was measured. The GSH concentration was calculated according to the standard sample and expressed as milligram GSH per mg of protein (mg/mgprot).

#### 2.7.4 SOD analysis

The reaction mixture was prepared according to the manufacturer’s instructions (catalogue no. A001-1-2, Jiancheng Bioengineering Institute, Nanjing, China) and the absorbance at 550 nm was measured. The SOD concentration was calculated according to the standard sample and expressed as U per mg of protein (U/mgprot).

### 2.8 Fe^2+^ assay

The content of Fe^2+^ was measured according to the manufacturer’s instructions (catalogue no. E-BC-K880-M, Elabscience Biotechnology, Wuhan, China). A total of 300 μL of the supernatant was mixed with 150 μL of reagent 2 and incubated at 37°C for 10 min. Then, the OD value at 520 nm was determined. The Fe^2+^ level is calculated as μmol per mg protein and expressed as a fold change versus the sham group.

### 2.9 Perls’ staining

Perls’ staining was performed according to the manufacturer’s instructions (Sangon Biotech, Shanghai, China). The sections were stained with Prussia blue working solution for 20 min, soaked in distilled water for 5 min and stained with 0.25% eosin for 15–30 s at room temperature. After being rinsed with tap water for 5 s, the slides were dehydrated, cleared, and sealed.

### 2.10 Transmission electron microscopy

The brain was fixed with 3% glutaraldehyde, dehydrated with alcohol, and embedded in Epon Resin 618. Sections with a thickness of 50 nm were stained with uranyl acetate and lead citrate. The morphology of the mitochondria was observed via an HT7700 transmission electron microscope (Hitachi High-Technologies).

### 2.11 Western blotting

Protein was extracted and quantified with a BCA protein assay kit (Beyotime, Shanghai, China). Proteins were separated by SDS‒PAGE electrophoresis and transferred to nitrocellulose membranes. The membrane was blocked with 5% nonfat milk at room temperature for 1 h and incubated with primary antibody overnight at 4°C. The membrane was incubated with an HRP-labeled secondary antibody at room temperature for 1 h. The bands were developed and analyzed. The primary antibodies used included antibodies against NRF2 (1:500, ab313825, Abcam, United States), FTL (1:500, ab75973, Abcam, United States), SLC7A11 (1:1,000, ab307601, Abcam, United States), GPX4 (1:1,000, ab125066, Abcam, United States), and β-actin (1:4,000, ab7817, Abcam, United States).

### 2.12 Immunohistochemistry

The sections were incubated with an anti-GFAP antibody (1:300, ab68428, Abcam, United States) and an anti-Iba-1 antibody (1:250, ab178846, Abcam, United States) at 4°C overnight and subsequently incubated with a secondary antibody at room temperature for 1 h. Finally, the sections were scanned with a Panoramic 250 FLASH II digital slide scanner.

### 2.13 ELISA

The levels of cytokines, including IL-6 (catalogue no. D731010), IL-1β (catalogue no. D731007), and TNF-α (catalogue no. D731168), were analyzed by ELISA kits (Sangon Biotech, Shanghai, China) according to the manufacturer’s instructions. The OD value at 450 nm was measured with a microplate reader. The concentration of cytokines was calculated according to the standard curve.

### 2.14 Statistical analysis

The data were statistically analyzed using Prism 8.0 software and expressed as the means ± SEMs. The Shapiro-Wilk test was used to test the normality of the data. The Bartlett’s test was used to test the homogeneity of variance. Multiple comparisons analyzed using ANOVA with Tukey’s *post hoc* test. *P <* 0.05 was considered significant.

## 3 Results

### 3.1 MGF improves neurological function and reduces the infarct area in stroke rats

**FIGURE 1:**
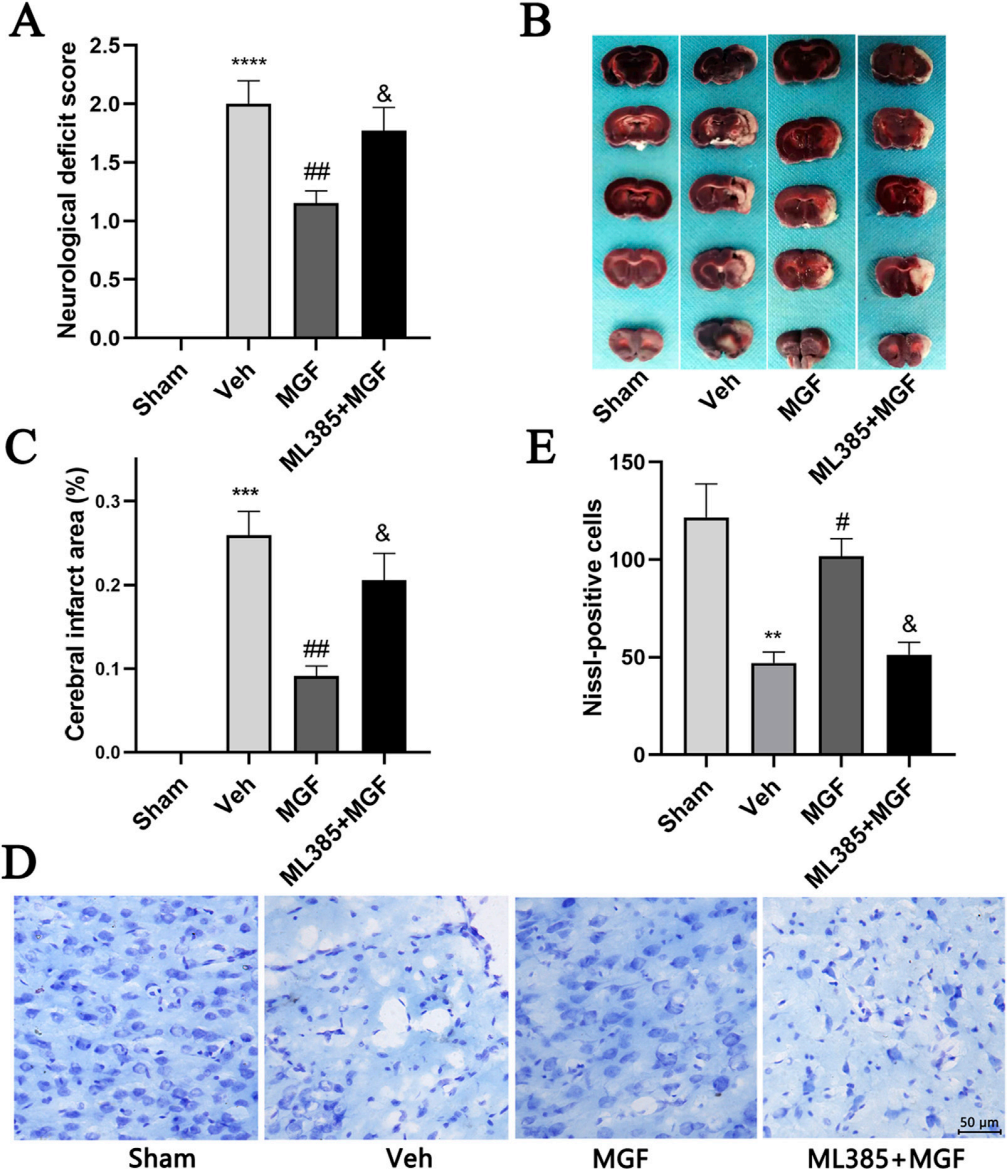
MGF decreases the neurological score and infarct area and protects neurons in stroke rats. **(A)** Neurological function was evaluated by neurological scoring (n = 13). **(B)** Representative images of TTC staining. **(C)** Quantification of the percentages of the infarct area (n = 6). **(D)** Representative images of Nissl staining (scale bar = 50 μm). **(E)** Quantitative analysis of Nissl-positive cells (n = 3). The data are presented as the means±SEMs and analyzed by one-way ANOVA followed by Tukey’s *post hoc* test; ^**^ *P* < 0.01, ^***^ *P* < 0.001, ^****^ *P* < 0.0001 vs. the sham group, ^#^ *P* < 0.05, ^##^ *P* < 0.01 vs. the veh group, ^&^ *P* < 0.05 vs. the MGF group.

The neurological score in the veh group was significantly greater (*P* < 0.0001 vs. sham) and was significantly reduced by MGF (*P* < 0.01 vs. veh), the effect of which was significantly reversed by ML385 intervention (*P* < 0.05 vs. MGF) (Figure 1A). TTC staining revealed that there were obvious infarct areas in the right cortex of stroke rats (*P* < 0.001 vs. sham). MGF significantly reduced the infarct size (*P* < 0.01 vs. veh). However, the effect of MGF was significantly reversed by ML385 intervention (*P* < 0.05 vs. MGF) (Figures 1B,C).

### 3.2 MGF restores the morphology of neurons and increases the number of neurons in stroke rats

Nissl staining revealed that the neurons in the veh group were atrophied, the nuclei were shrunken, and the number of Nissl-positive cells was significantly lower (*P* < 001 vs. sham). MGF significantly restored neuronal morphology and increased the number of Nissl-positive cells (*P* < 005 vs. veh), which were dramatically reversed by ML385 intervention (Figures 1D,E).

### 3.3 MGF enhances behavioral performance in stroke rats

**FIGURE 2:**
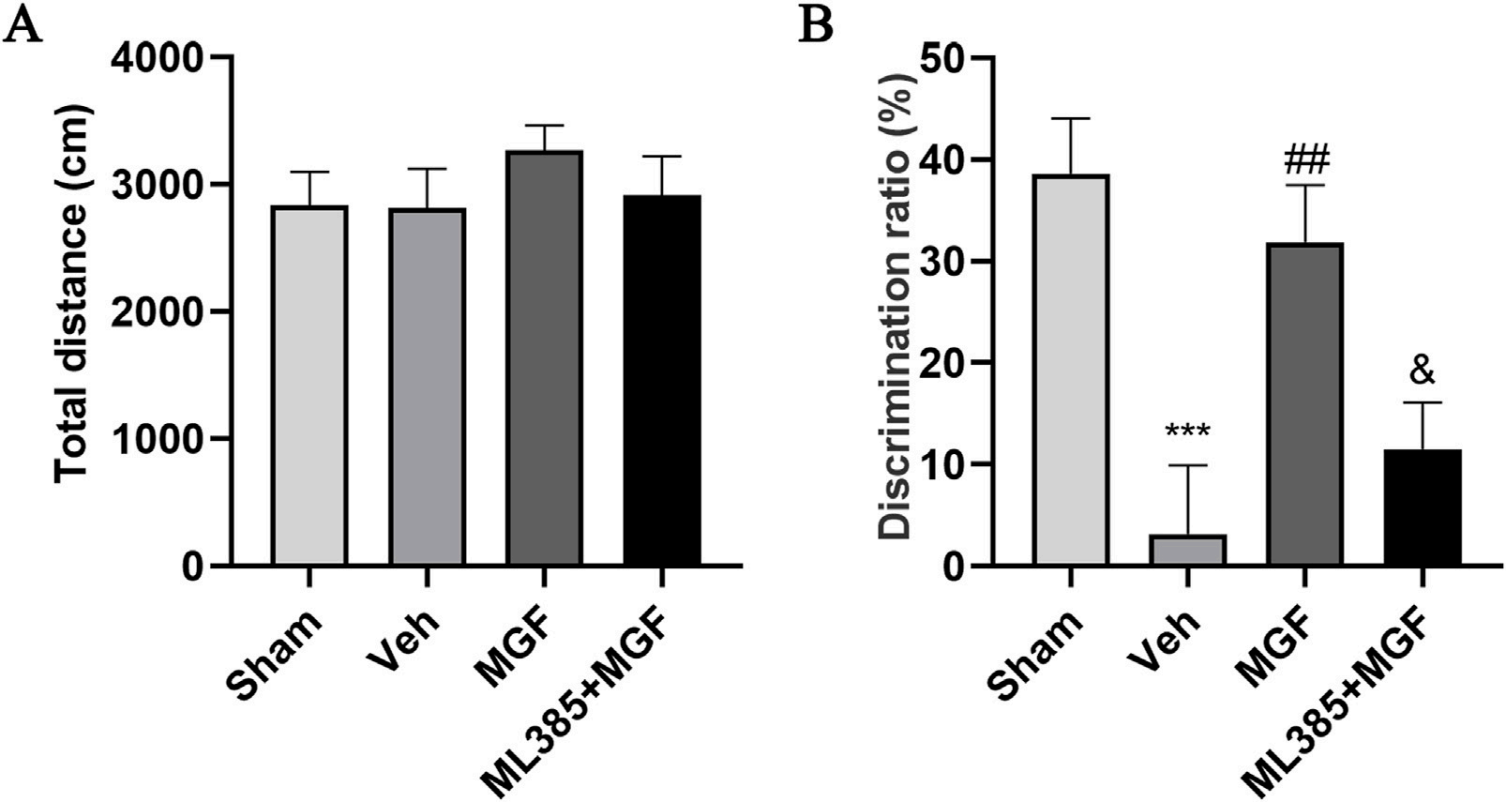
MGF improves learning and memory ability in stroke rats. **(A)** The open field test was used to evaluate motor ability. **(B)** A novel object recognition test was adopted to evaluate learning and memory ability. The data are presented as the means±SEMs and analyzed by one-way ANOVA followed by Tukey’s *post hoc* test; ^***^ *P* < 0.001 vs. the sham group; ^##^ *P* < 0.01 vs. the veh group; ^&^ *P* < 0.05 vs. the MGF group. n = 12–15.

The rats that cannot move were excluded from behavioral tests. In addition, total movement distance was evaluated to ensure the consistency of animal motor ability by open field test. As shown in Figure 2A, no significant difference was observed in the total movement distance among the three groups (Figure 2A), ensuring that the learning and memory ability of rats is not caused by motor disability (Zhang et al., 2023). The data by the novel object recognition test revealed that the discrimination ratio of the rats in the veh group was significantly lower (*P* < 0.001 vs. sham) and was significantly increased by MGF (*P* < 0.01 vs. veh). However, ML385 significantly inhibited the effects of MGF on the discrimination ratio (*P* < 0.05 vs. MGF) (Figure 2B).

### 3.4 MGF alleviates oxidative stress in stroke rats

**FIGURE 3:**
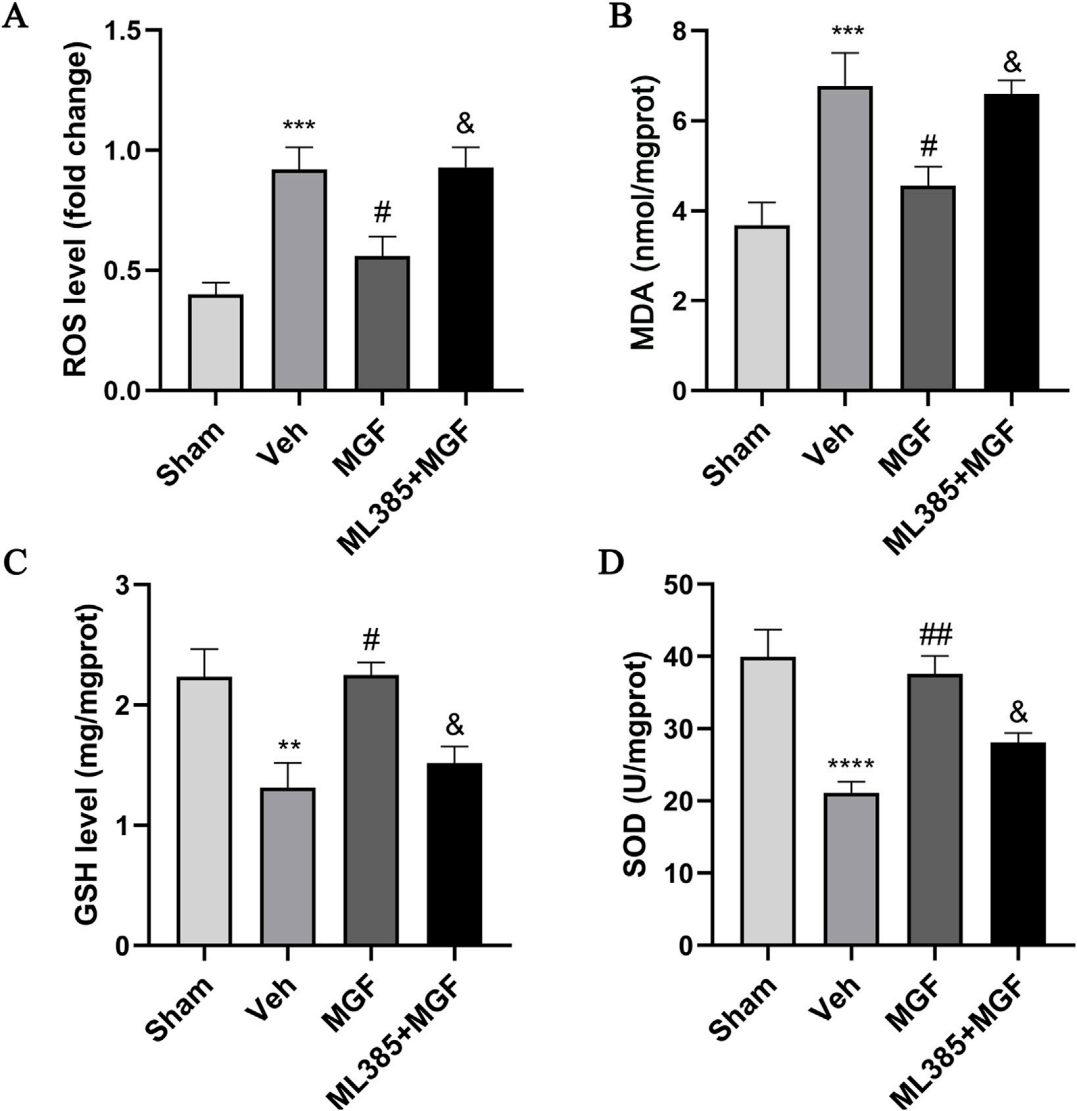
MGF alleviates oxidative stress in stroke rats. Quantitative analyses of ROS **(A)**, MDA **(B)**, GSH **(C)**, and SOD **(D)** levels. The data are presented as the means±SEMs and analyzed by one-way ANOVA followed by Tukey’s *post hoc* test; ^**^ *P* < 0.01, ^***^ *P* < 0.001, ^****^ *P* < 0.0001 vs. the sham group; ^#^ *P* < 0.05, ^##^ *P* < 0.01 vs. the veh group; ^&^ *P* < 0.05 vs. the MGF group. n = 7–9.

As shown in Figure 3, the levels of ROS and MDA in the veh group were significantly greater (all *P* < 0.001 vs. sham), and the levels of GSH and SOD were significantly lower (*P* < 0.01, *P* < 0.0001 vs. sham). MGF significantly decreased the levels of ROS and MDA (all *P* < 0.05 vs. veh) and increased the levels of GSH and SOD (*P* < 0.05, *P* < 0.01 vs. veh), which were significantly reversed by ML385 (all *P* < 0.05 vs. MGF).

### 3.5 MGF attenuates neuroinflammation in stroke rats

**FIGURE 4:**
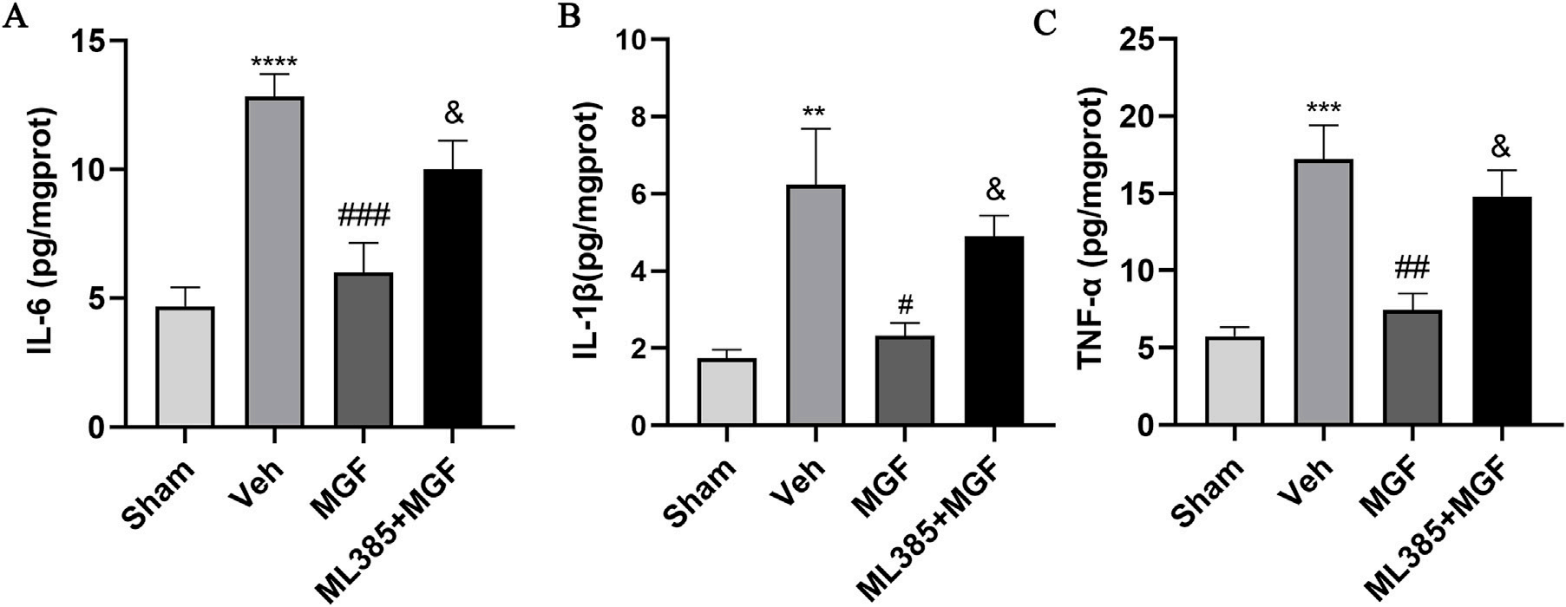
MGF attenuates neuroinflammation in stroke rats. Quantitative analyses of IL-6 **(A)**, IL-1β **(B)**, and TNF-α **(C)** levels. The data are presented as the means±SEMs and analyzed by one-way ANOVA followed by Tukey’s *post hoc* test; ^**^ *P* < 0.01, ^***^ *P* < 0.001 vs. the sham group; ^#^ *P* < 0.05, ^##^ *P* < 0.01, ^###^ *P* < 0.001 vs. the veh group; ^&^ *P* < 0.05 vs. the MGF group. n = 6.

As shown in Figure 4, the levels of IL-6, IL-1β and TNF-α in the brain tissue of the rats in the veh group were significantly greater (*P* < 0.0001, *P* < 0.01, *P* < 0.001 vs. sham) and were significantly lower in the MGF group (*P* < 0.001, *P* < 0.05, *P* < 0.01 vs. veh). ML385 significantly reversed the inhibitory effects of MGF on cytokines (all *P* < 0.05 vs. MGF).

### 3.6 MGF suppresses ferroptosis in stroke rats by activation of the NRF2/ARE pathway

**FIGURE 5:**
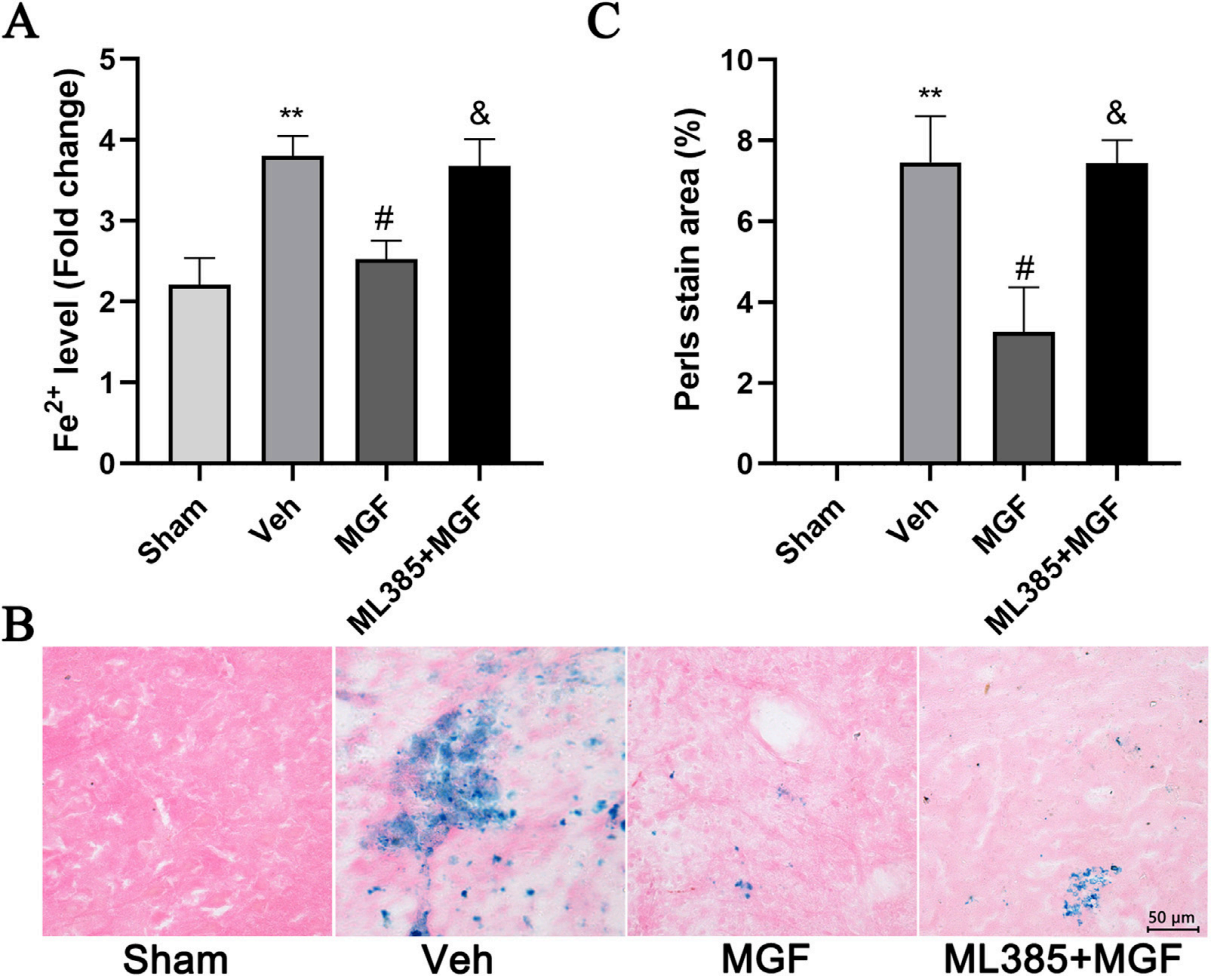
MGF suppresses iron levels in stroke rats. **(A)** Fe^2+^ levels in cerebral tissue (n = 9). **(B)** Representative images of Perls’ stain (scale bar = 50 μm). **(C)** Quantitative analysis of the Perls’ staining area (n = 3). The data are presented as the means±SEMs and analyzed by one-way ANOVA followed by Tukey’s *post hoc* test; ^**^ *P* < 0.01 vs. the sham group; ^#^ *P* < 0.05 vs. the veh group; ^&^ *P* < 0.05 vs. the MGF group.

**FIGURE 6:**
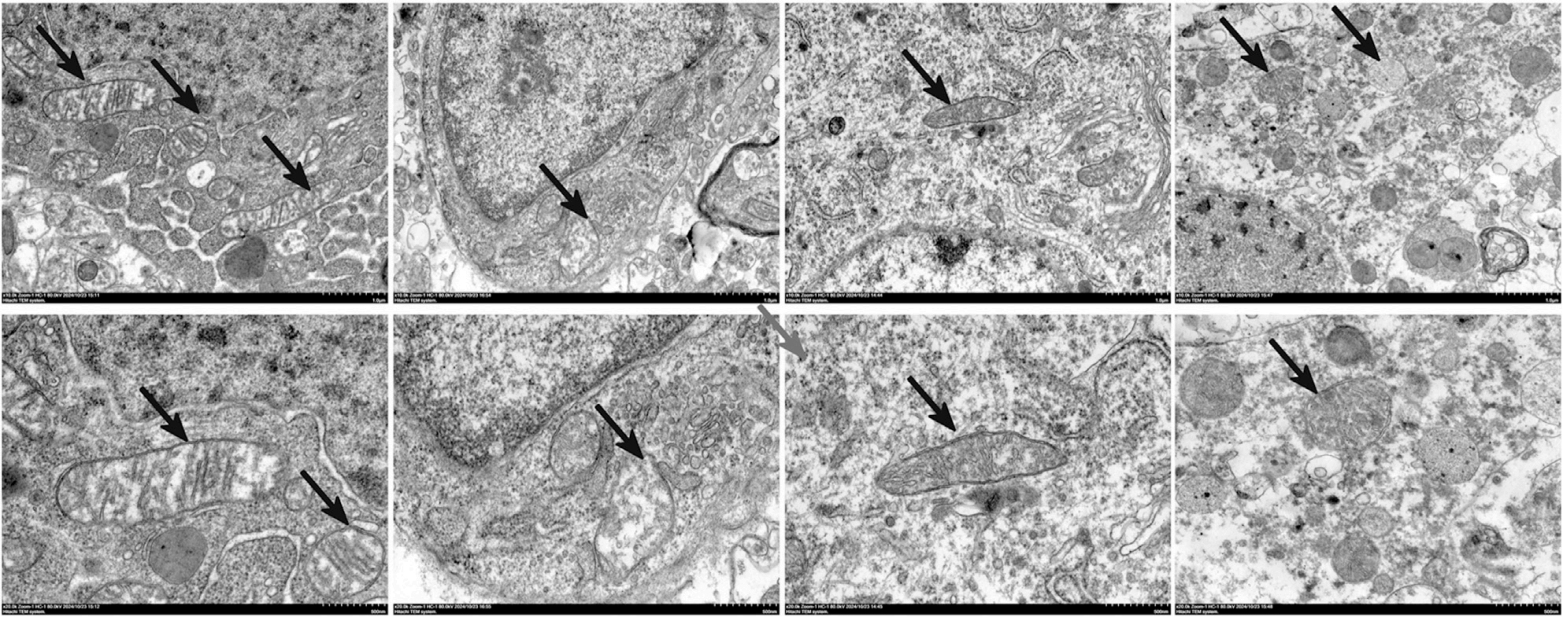
Transmission electron microscopy analysis of the restoration of mitochondrial morphology by MGF in stroke rats. Scale bar = 500 nm, n = 3.

**FIGURE 7:**
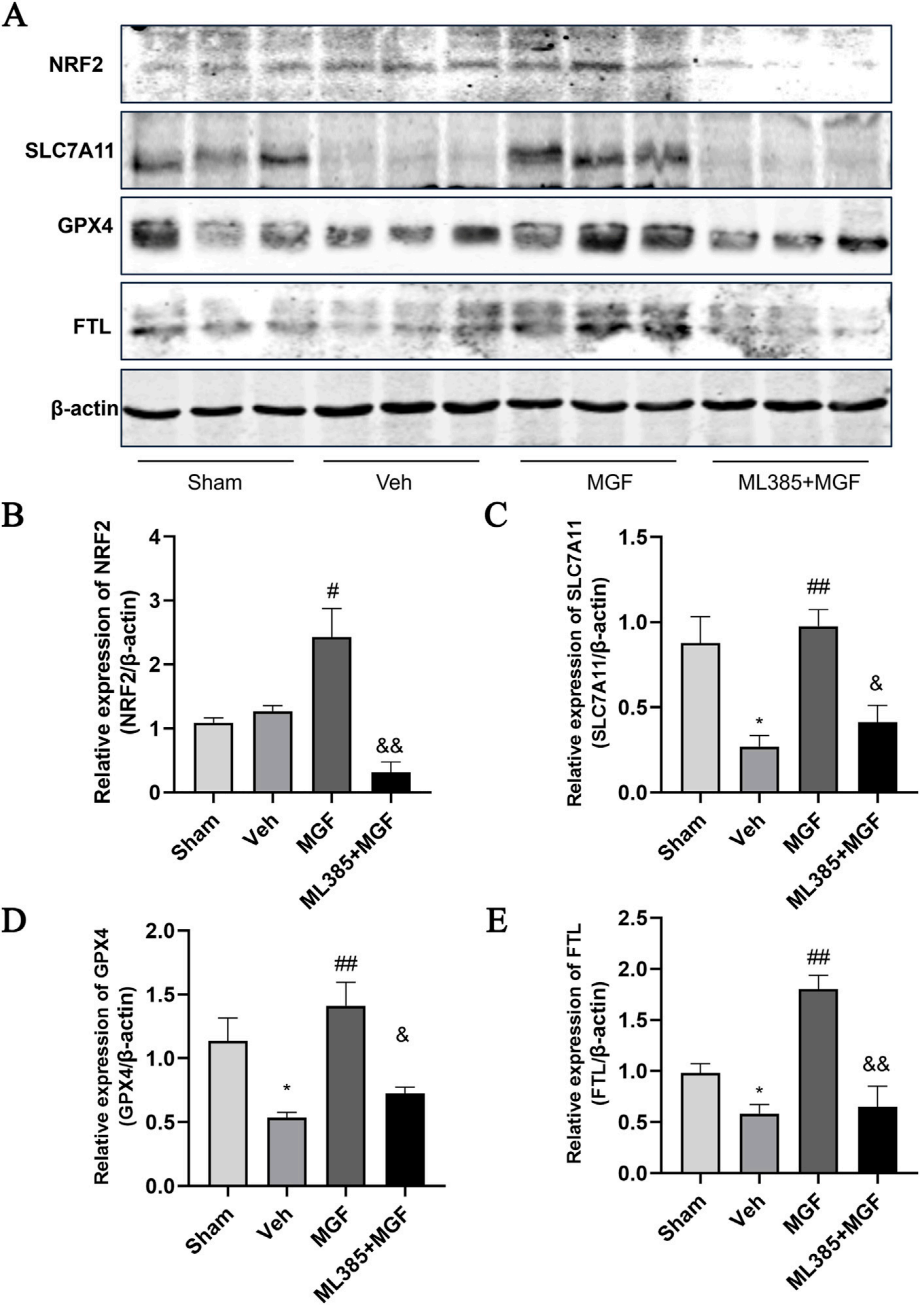
The expression of NRF2/ARE pathway. **(A)** Representative images of western blots. **(B)** Quantification of the relative expression of NRF2 **(B)**, SLC7A11 **(C)**, GPX4 **(D)**, and FTL **(E)**. The data are presented as the means±SEMs and analyzed by one-way ANOVA followed by Tukey’s *post hoc* test; ^*^ *P* < 0.05 vs. the sham group, ^##^ *P* < 0.01 vs. the veh group, ^&^ *P* < 0.05 vs. the MGF group. n = 3.

The Fe^2+^ level in the veh group was significantly greater (*P* < 0.01 vs. sham) and was significantly reduced by MGF (*P* < 0.05 vs. veh). ML385 significantly inhibited the effect of MGF on the Fe^2+^ level (*P* < 0.05 vs. MGF) (Figure 5A). Consistent with these results, the results of the Perls’ stain analysis revealed that the Perls’ stain area in the veh group was significantly greater (*P* < 0.01 vs. sham) and was significantly decreased by MGF (*P* < 0.05 vs. veh). ML385 markedly reversed the inhibitory effect of MGF on the Perls’ stain area (*P* < 0.05 vs. MGF) (Figures 5B,C). Furthermore, the ultrastructure of the mitochondria was analyzed by transmission electron microscopy. Compared with those in the sham group, the rats in the veh group presented an obviously decreased mitochondrial ridge and increased mitochondrial membrane density. MGF obviously recovered the mitochondrial morphology. In contrast, ML385 significantly reversed the effect of MGF on mitochondrial morphology (Figure 6). Western blot analysis revealed that cerebral ischemia marginally induced the expression of NRF2 and significantly decreased the expression of SLC7A11, GPX4, and FTL (*P* < 0.05 vs. sham). MGF significantly upregulated the expression of NRF2 (*P* < 0.05 vs. veh), SLC7A11 (*P* < 0.01 vs. veh), GPX4 (*P* < 0.01 vs. veh), and FTL (*P* < 0.01 vs. veh). However, ML385 significantly reversed the influence of MGF on these proteins (Figure 7). The band density was quantified by ImageJ (NIH, United States). The relative expression levels of the proteins were normalized to β-actin.

## 4 Discussion

Previous studies have shown that MGF has various pharmacological activities, including anti-inflammatory, antioxidative stress, antitumor, antidiabetes, immune regulatory effects, and can be used for the treatment of diseases in the cardiovascular and cerebrovascular systems, central nervous system, respiratory system, and other diseases (Du et al., 2018). However, whether MGF has a protective effect on stroke and its mechanism of action remain unclear. Recently, ferroptosis has been proposed to play an important role in the pathogenesis of neurological injury after stroke (Cui et al., 2021). NRF2 is an important regulator of cellular and systemic defense against both endogenous and exogenous stress and regulates various cellular protective genes to inhibit ferroptosis (Anandhan et al., 2023). Activating NRF2 to inhibit ferroptosis has great potential in the treatment of ischemic stroke. It has been demonstrated that MGF protects neurological function primarily by activating NRF2 (Dang et al., 2022).

In our study, the neuroprotective effect of MGF on a stroke model and the underlying mechanism were investigated. The data showed that MGF strikingly reduced the neurological score, indicating that MGF effectively alleviated the neurological deficits after stroke. The TTC results revealed that MGF significantly reduced the size of the cerebral infarct area, which suggests that MGF intervention can help attenuate brain tissue damage. In addition, Nissl staining revealed that MGF significantly restored the morphology of neurons and increased the number of neurons in the Veh group. These results indicate that MGF can effectively alleviate neuronal injury. The incidence of cognitive dysfunction caused by ischemic stroke is approximately 30%, and it manifests mainly as learning and memory impairment, which seriously affects the prognosis of patients (Pulvermüller et al., 2021). It has been previously shown that MGF can improve the learning and memory ability of animal models of various nervous system diseases (Zhang et al., 2024). In this study, through a novel object recognition test, we found that MGF can significantly attenuate learning and memory dysfunction instroke rats.

Oxidative stress and neuroinflammation are the main pathological changes associated with ischemic stroke. MGF has antioxidative effects, inhibits neuroinflammatory damage and improves mitochondrial function in nervous system diseases (Lei et al., 2021). Our study revealed that MGF significantly inhibited the levels of inflammatory factors (IL-6, IL-1β and TNF-α) and the levels of the oxidative stress factors ROS and MDA, and significantly increased the levels of the antioxidant stress factors GSH and SOD, indicating that MGF can effectively alleviate the oxidative stress and neuroinflammation induced by MCAO. In addition, the effects of MGF was inhibited by ML385, suggesting that MGF might suppress oxidative stress and neuroinflammation by activating NRF2.

Ferroptosis is a newly described type of cell death induced by the small molecule erastin, or RSL3, discovered in 2012. Unlike other types of programmed cell death, ferroptosis is iron-dependent and has unique mitochondrial morphology and bioenergetic characteristics (Stockwell, 2022). It has been demonstrated that ferroptosis participates in the physiological and pathological process of stroke injury. In animal models, ferroptosis inhibitors such as iron chelating agents, liproxstatin-1 and ferrostatin-1 effectively reduce reperfusion injury after ischemic stroke and improve neurological prognosis (Guo et al., 2023). These findings suggest that inhibiting ferroptosis in nerve cells may be an effective means to treat ischemic stroke. In this study, we found that MGF significantly reduced Fe^2+^ levels in brain tissue and iron deposition in brain tissue. The main characteristic of ferroptosis is mitochondrial morphological changes, including changes in the mitochondrial membrane density concentration, volume reduction, mitochondrial ridge reduction or disappearance, and outer membrane rupture (Gao et al., 2019). The mitochondria of nerve cells from stroke rats exhibited a clear ferroptosis-like morphology, and MGF ameliorated morphological abnormalities consistent with ferroptosis.

System X_c_^−^/GPX4 inhibits ferroptosis by reducing the accumulation of lipid peroxides. System X_C_^−^ can reversely transport Cys and Glu in a 1:1 manner and catalyze the conversion of Cys into GSH. As a key subunit of System X_C_^−^, SLC7A11 is essential for the synthesis of GSH, and the inhibition of SLC7A11 disrupts cellular redox homeostasis and leads to ferroptosis (Jiang et al., 2023). GPX4 is another negative regulator of ferroptosis. GPX4 can degrade small molecule peroxides and lipid peroxides and inhibit lipid peroxidation (Liu Y. et al., 2023). GSH is the main substrate of GPX4, and the decreased GPX4 activity caused by GSH consumption is unable to eliminate harmful lipid peroxides. Knocking down GPX4 can lead to serious deficits in spatial learning and memory, hippocampal neurodegeneration, lipid peroxidation, oxidative stress and neuroinflammation in mice. Ferroptosis inhibitors can effectively alleviate these symptoms (Hambright et al., 2017).

FTL is mainly responsible for the storage of free iron ions in cells, and decrease in iron storage capacity leads to the accumulation of iron ions in the body and the generation of too many oxygen free radicals through the Fenton reaction, resulting in ferroptosis (Fujimaki et al., 2019; Dowdle et al., 2014). It has been shown that NRF2 is involved in the regulation of iron homeostasis by activating FTL expression (Koorts and Viljoen, 2007). To clarify the mechanism by which MGF inhibits ferroptosis, we analyzed the expression of NRF2 and downstream ferroptosis-related proteins by Western blotting. The data revealed that MGF significantly upregulated the expression of NRF2, GPX4, SLC7A11 and FTL, whereas ML385 inhibited the influence of MGF on these proteins, suggesting that MGF might inhibits ferroptosis in stroke by targeting the NRF2/ARE pathway. In conclusion, this study suggests that MGF plays a role in protecting neurological function in stroke and that the NRF2/ARE pathway is a potential target of MGF. This protective effect may be related to the inhibition of oxidative stress, neuroinflammation and ferroptosis. However, several limitations exist in our study. (1) The neuroprotective effect of MGF on female rats remains to be explored. (2) Our study lacks the time-course data, such as neurological scoring, TTC staining, and the expression level of the NRF/ARE pathway. (3) A ML385-only group is lacked in the present study. Our future study will add a ML385 group to further confirm the specific interaction between MGF and NRF2. (4) Although our study confirmed the regulatory effect of MGF on the NRF/ARE pathway, the exact mechanism by which MGF interacts with NRF2 is still to be clarified at *in vitro* neuronal cell level. (5) Because MGF is a multitarget, multipathway drug, a single mechanism, such as NRF2/ARE, cannot fully explain the neuroprotective effect of MGF on stroke. Transcriptomic or proteomic analysis will contribute to a more comprehensive understanding of the mechanism of MGF neuroprotection. Our future work will elaborate on the in-depth mechanism by which MGF acts on the upstream targets of NRF2 through omics-based approaches and NRF2 overexpression/knockout models.

## 5 Conclusion

This study demonstrates that MGF ameliorates neurological deficits and ferroptosis via NRF2/ARE activation in ischemic stroke rats. These findings provide a potential therapeutic rationale for MGF in stroke management.

## Data Availability

The original contributions presented in the study are included in the article/Supplementary Material, further inquiries can be directed to the corresponding author.
